# Thymic large cell neuroendocrine carcinoma: report of a resected case - a case report

**DOI:** 10.1186/1749-8090-5-115

**Published:** 2010-11-22

**Authors:** Fumihiro Ogawa, Akira Iyoda, Hideki Amano, Kenji Nezu, Shi-Xu Jiang, Isao Okayasu, Yukitoshi Satoh

**Affiliations:** 1 Department of Thoracic Surgery, Kitasato University School of Medicine, Kanagawa, Japan; 2 Department of Pathology, Kitasato University School of Medicine, Kanagawa, Japan

## Abstract

Thymic large cell neuroendocrine carcinomas (LCNECs) are very rare. We here describe a case in which the tumor could be completely resected. A 55-year-old male was admitted to our hospital for treatment of an anterior mediastinal tumor found at a regular health check-up. The patient underwent an extended thymectomy of an invasive thymoma of Masaoka's stage II that had been suspected preoperatively. The tumor was located in the right lobe of the thymus and was completely resected. Final pathological diagnosis of the surgical specimen was thymic LCNEC. The patient underwent adjuvant chemotherapy with irinotecan and cisplatin in accordance with the diagnosis of a lung LCNEC, and is alive without recurrence or metastasis 16 months after surgery.

## Background

Primary thymic neuroendocrine carcinomas (NECs) were categorized under the rubric of 'thymomas' until 1972, when Rosai and Higa suggested that these tumors were sufficiently distinctive to warrant classification as carcinoid tumors [1]. Thymic NECs are relatively rare neoplasms that account for only approximately 2% to 4% of all anterior mediastinal neoplasms [2]. In 1999, the World Health Organization established thymic epithelial tumor criteria and reclassified thymic carcinoma, referring to NECs as a subtype [3]. In particular, the LCNEC was subclassified in the thymic NECs in accordance with the classification of pulmonary neuroendocrine tumors. Detailed clinical features of thymic LCNECs are still unknown, however, because of their rareness. We described a case with a review of the literature, focusing on the most likely optimal treatment it.

## Case presentation

**Figure 1:**
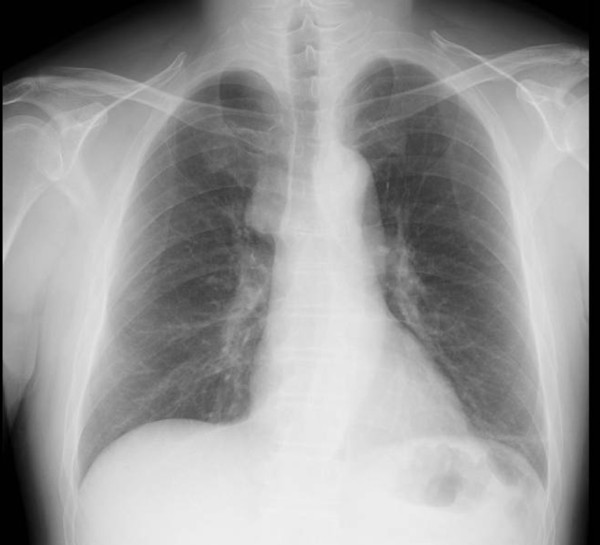
**Chest x-ray showing a solid mass with a clear border at the right hilum and a negative silhouette sign for the right first arch**.

**Figure 2:**
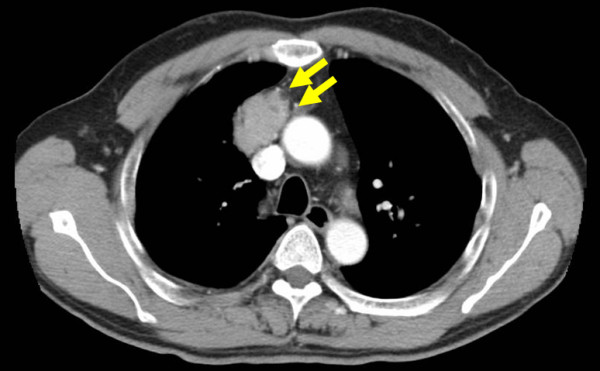
**Enhanced chest CT scan revealing a 42-mm-sized solid mass with an unclear margin (arrows) with the normal thymus in the anterior mediastinum**.

**Figure 3:**
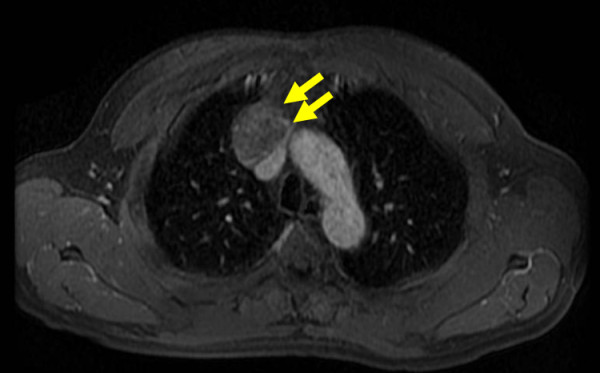
**Chest MRI using intravenous contrast medium showed iso-intensity of the mass on a T1-weighted image**.

**Figure 4:**
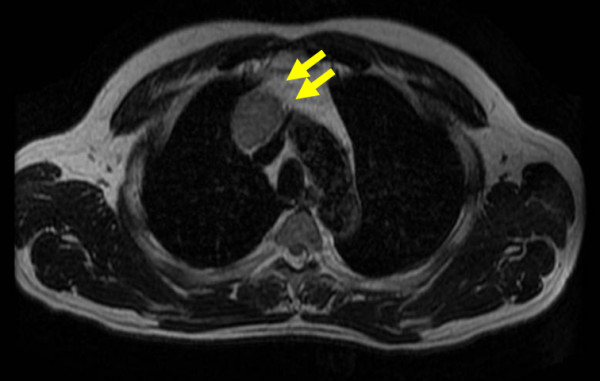
**Chest MRI using intravenous contrast medium showed iso-intensity of the mass on a T2-weighted image with an unclear rim (arrows), as with the chest CT, too**.

A 55-year-old Japanese male was admitted to the Kitasato University Hospital for further examination and treatment for an abnormal shadow on the chest x-ray found at a regular health check-up. He had smoked 35 packs per year for 20 years. Chest x-ray films showed a solid mass with a clear border at the right hilum and a negative silhouette sign for the right first arch (Figure 1). Enhanced chest computed tomography (CT) revealed a solid mass 42 mm in diameter with a partially unclear margin with the normal thymic tissue in the anterior mediastinum (Figure 2). Magnetic resonance imaging (MRI) using intravenous contrast medium showed isointensity of the mass on both T1- and T2-weighted images (Figure 3, 4). Although chest CT and MRI revealed no invasion of the superior vena cava and the innominate vein, the tumor was highly suspected to have invaded the normal thymic tissue. Laboratory findings and results for tumor markers such as CEA (carcinoembryonic antigen), NSE (neuron specific enolase), and ProGRP (pro-gastrin releasing peptide) were all within normal ranges, preoperatively.

Under the diagnosis of invasive thymoma or thymic carcinoid, the patient underwent an extended thymectomy. The tumor was intraoperatively revealed in the right lobe of the thymus without any invasion to the adjacent organs:the aorta, superior vena cava, pericardium, bilateral phrenic nerve, or the right lung. Because the tumor had invaded the right parietal pleura, we also resected the right parietal pleura with a sufficient surgical margin.

Macroscopically, the elastic soft tumor surrounded by thymic fat tissue was 40 × 35 × 28 mm in size. The cut surface was mainly whitish-yellow in color and showed focal necrosis and red bloody spots.

**Figure 5:**
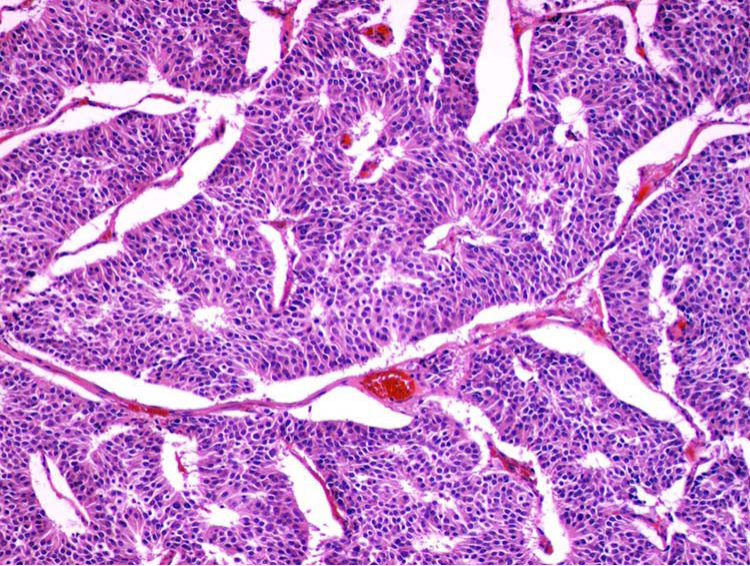
**The tumor cells were arranged in wide trabeculae with irregular nests separated by thin fibrovascular stroma, and scattered abortive rosette-like structures were encountered**. (hematoxylin and eosin staining, ×40).

**Figure 6:**
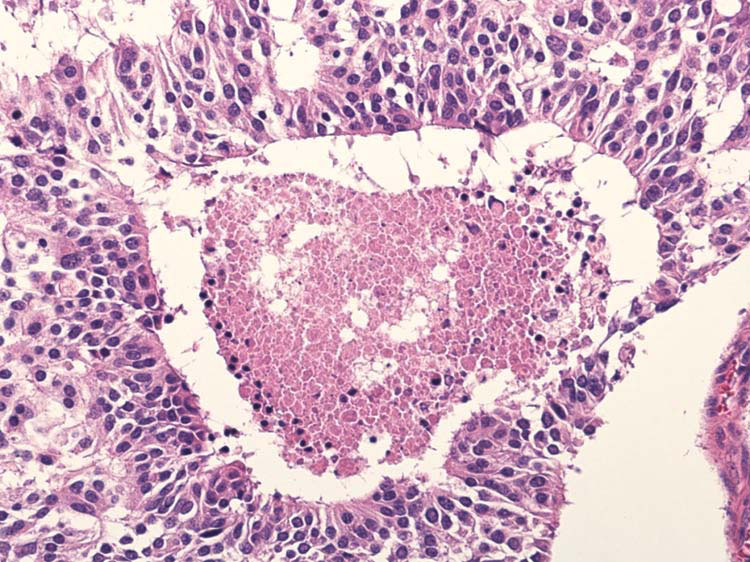
**Small foci of coagulative necrosis were also observed**. (hematoxylin and eosin staining, ×100).

**Figure 7:**
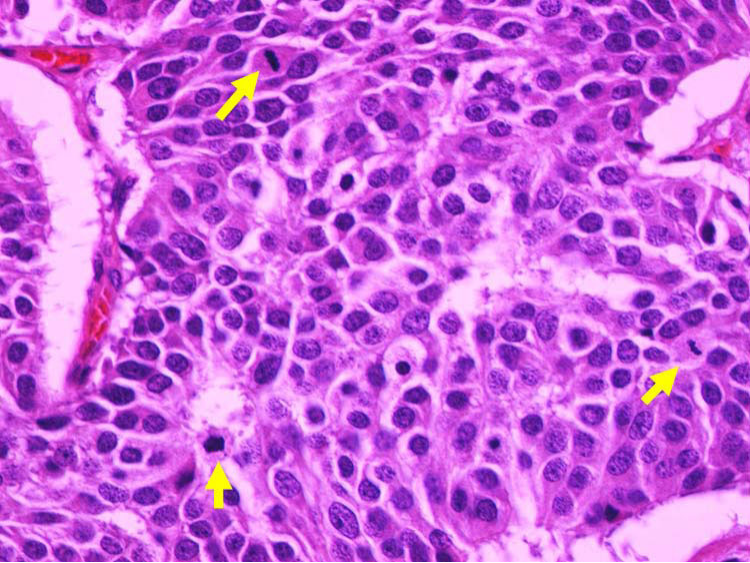
**Mitosis(arrows) counts ranged around 30 per 10 high-power fields**. (hematoxylin and eosin staining, ×400).

**Figure 8:**
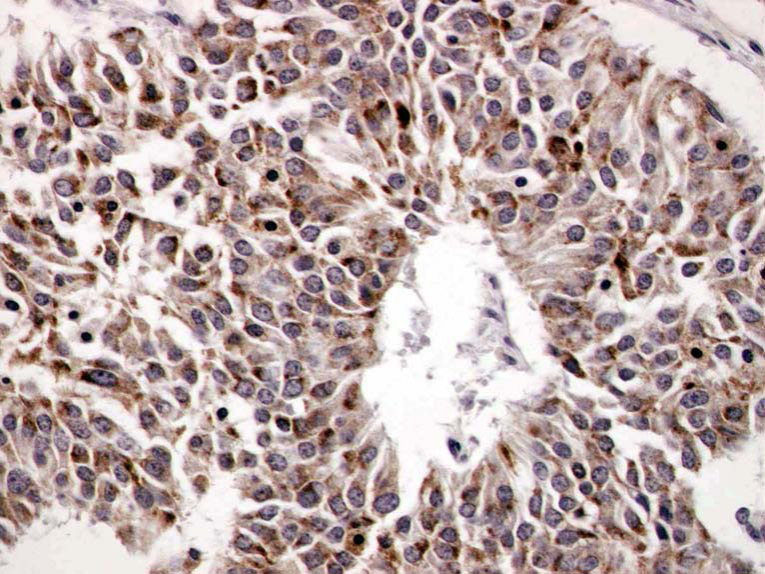
**Tumor cells were diffusely positive for chromogranin A (×400)**.

Microscopically, the tumor manifested morphologic features of a carcinoid. The tumor cells were arranged in wide trabeculae with irregular nests separated by thin fibrovascular stroma, and scattered abortive rosette-like structures (Figure 5). The tumor cells were oval to polygonal in shape with abundant eosinophilic and granular cytoplasm. The nuclear chromatin was granular and the nucleoli were inconspicuous. Small foci of coagulative necrosis were also observed (Figure 6). The average mitotic count was 30 per each of 10 high-power fields (Figure 7), and the Ki-67 indices using MIB-1 immunohistochemical staining ranged from 20% to 30%. Immunohistochemically, the tumor cells were diffusely positive for chromogranin A (Figure 8), synaptophysin, and neural cell adhesion molecule (NCAM), confirming a neuroendocrine nature. Thus, the final pathological diagnosis of thymic LCNEC was made. The tumor also invaded atrophic normal thymic tissue.

The patient underwent adjuvant chemotherapy based on a platinum doublet containing cisplatin at 60 mg/m^2 ^and irinotecan at 60 mg/m^2 ^for three courses, and is alive without recurrence or metastasis at 16 months after surgery.

## Discussion

The neuroendocrine subtype of thymus tumors is defined on the basis of histopathological features and immunophenotypes. In recent studies [4,5], NECs have been morphologically categorized into four main types: typical carcinoid, atypical carcinoid, LCNEC, and small-cell carcinoma. To our knowledge, LCNECs and small-cell carcinomas are highly malignant and have a poorer prognosis than do other thymic epithelial tumors. The LCNEC is included as a separate entity because of differences from carcinoids in survival rates as well as its incidence and clinical, epidemiologic, histological, and molecular characteristics.

Although chest CT and MRI revealed no invasion to the superior vena cava or the innominate vein in the present case, and T1- and T2-weighted images demonstrated isointensity, the tumor was highly suspected of having invaded the normal thymic tissue due to its unclear rim. Therefore, our preoperative diagnosis was an invasive thymoma or a carcinoid.

For optimal treatment, an accurate pretherapeutic diagnosis is important. However, as a thymic tumor is not always morphologically homogeneous, this may be difficult with a standard needle biopsy. Surgery offers the best chance for a definitive diagnosis and curative treatment of thymic tumors. The differential diagnosis for the anterior mediastinum includes other primary mediastinal tumors, mainly thymoma, paraganglioma, lymphoma, parathyroid adenoma or carcinoma, as well as medullary carcinoma of the thyroid. The most difficult but most important differential diagnosis in this setting is with thymoma, particularly of the spindle cell type. This latter can often show areas displaying a prominent neuroendocrine appearance with abundant epithelial cells disposed radially around an empty space closely simulating the microacinar growth pattern sometimes observed in carcinoids. To make a successful differential diagnosis, immunohistochemical staining can be helpful. Even though both types of lesions share strong CAM 5.2 positivity, thymomas are negative for neuroendocrine markers (e.g. chromogranin A, synaptophysin, NCAM, and CD56) and may be useful for NECs [6].

Thymic LCNEC is very rare. A search of the PubMed database revealed only a few case reports in the literature [7-11]. Mega et al. reviewed 10 cases of thymic LCNECs in Japan [7]. As seen in Table 1, surgical resection was performed in 8 of the 10 cases, but most of the patients were at an advanced stage of disease and half had recurrence. Furthermore, recurrence occurred relatively soon after surgery (range 2 to 7 months) and their prognoses were very poor. Cesar et al. reported [6], the primary mediastinal NEC to represent a separate biologic entity from carcinoids arising at other locations, with disease-free survivals of 50% at 5 years and 9% at 10 years for well differentiated tumors (i.e. typical carcinoids), 20% at 5 years and 0% at 10 years for moderately differentiated tumors (i.e. atypical carcinoids), and 0% at 5 years for poorly differentiated tumors. Therefore, it can be considered that a well differentiated grade and complete surgical removal followed by adjuvant therapy offer curative potential and are significant factors for prolonged survival [7-9].

**Table 1:** The case report of thymic LCNECs in Japan

Case	Age	Gender	Report (year)	Size	Masaoka stage	Treatment	Prognosis
1	53	F	Kurashima (2002)	50	IVa	Op+Cx+Rx	N.S.
2	50	M	Miura (2003)	40	IVa	Op	N.S.
3	63	F	Hirami (2004)	50	IVb	Op+Rx	4 M Recurrence
4	57	F	Nagata (2005)	70	II	Op+Rx	7 M Recurrence
5	69	F	Kitagawa (2006)	N.S.	IVb	Cx	N.S.
6	N.S.	N.S.	Shimokawa (2006)	N.S.	N.S.	N.S.	N.S.
7	46	M	Tao (2006)	N.S.	IVb	Op+Cx+Rx	4 M Recurrence
8	63	F	Tao (2006)	N.S.	IVb	Op+Cx+Rx	4 M Recurrence
9	67	F	Kou (2007)	N.S.	IVa	Op+Cx+Rx	N.S.
10	67	F	Mega (2008)	60	IVb	Op+Cx+Rx	6 M Recurrence
11	55	M	This Case	42	II	Op+Cx	No Recurrence

Op: operation Cx: chemotherapy Rx: irradiation N.S.: Not shown

Currently, there is no evidence to support the use of postoperative therapy for Thymic LCNECs. Recent studies [12-14] of LCNEC of the lung recommended postoperative administration of adjuvant chemotherapy with platinum-based combination regimens (e.g. etoposide and others), which is the regimen for small cell lung carcinoma similar to the clinicopathologic and biologic features of LCNEC. Their results showed good prognosis. Platinum-based combination regimens were effective for the patients with LCNEC in their studies. Likewise, we believe that surgery and adjuvant therapy are needed to treat LCNEC in the thymus. Therefore we selected the regimen, cisplatin/irinotecan, for small cell lung carcinoma because Noda et al. revealed that cisplatin/irinotecan provided better results than did cisplatin/etoposide [15]. And Fujiwara et al. [16] also indicated that irinotecan-based regimens might be as active against LCNEC of the lung as against SCLC. Since recurrence of thymic LCNECs occurs within a short duration after surgery and their prognosis is very poor, we regarded this disease as having an extensive status at resection. Therefore, we selected the regimen, cisplatin/irinotecan. However, the odalities for adjuvant chemotherapy remain to be defined.

## Conclusion

Because primary thymic LCNECs are very rare, and the patients'prognoses are very poor, along with the lack of experience, a standardized treatment protocol, and the limited literature, all these contributing factors make it a difficult tumor to treat. Additional studies area warranted to determine the optimal treatment of thymic LCNECs.

## Competing interests

The authors declare that they have no competing interests.

## Authors' contributions

FO carried out the manuscript and collected references. YS coordinated all authors. FO and YS underwent this operation, and AI, HA, and KN helped for clinical support with them. SJ and IO reported pathological findings and took the pathologic pictures. AI and YS helped to draft the manuscript.

All authors read and approved the final manuscript.

## Consent

Written informed consent was obtained from patient for publication of this case report and any accompanying images. A copy of the written consent is available for review by the Editor-in-Chief of this journal.
